# Validation of a computer case definition for sudden cardiac death in opioid users

**DOI:** 10.1186/1756-0500-5-473

**Published:** 2012-08-31

**Authors:** Vivian K Kawai, Katherine T Murray, C Michael Stein, William O Cooper, David J Graham, Kathi Hall, Wayne A Ray

**Affiliations:** 1 Division of Pharmacoepidemiology, Department of Preventive Medicine, Nashville, TN, USA; 2 Departments of Medicine and Pharmacology, Divisions of Cardiology, Nashville, TN, USA; 3 Rheumatology (CMS) and, Nashville, TN, USA; 4 Clinical Pharmacology, Vanderbilt University, Nashville, TN, USA; 5 Department of Pediatrics, Nashville, TN, USA; 6 Geriatric Research, Education and Clinical Center, Nashville Veterans Administration Medical Center, Nashville, TN, USA; 7 Food and Drug Administration, Silver Spring, MD, USA; 8 Department of Preventive Medicine, Vanderbilt University, Village at Vanderbilt, Suite 2600, 1501 21st Ave South, Nashville, TN 37212, USA

**Keywords:** Case definition, Automated databases, Sudden cardiac death, Opioids, Propoxyphene

## Abstract

**Background:**

To facilitate the use of automated databases for studies of sudden cardiac death, we previously developed a computerized case definition that had a positive predictive value between 86% and 88%. However, the definition has not been specifically validated for prescription opioid users, for whom out-of-hospital overdose deaths may be difficult to distinguish from sudden cardiac death.

**Findings:**

We assembled a cohort of persons 30-74 years of age prescribed propoxyphene or hydrocodone who had no life-threatening non-cardiovascular illness, diagnosed drug abuse, residence in a nursing home in the past year, or hospital stay within the past 30 days. Medical records were sought for a sample of 140 cohort deaths within 30 days of a prescription fill meeting the computer case definition. Of the 140 sampled deaths, 81 were adjudicated; 73 (90%) were sudden cardiac deaths. Two deaths had possible opioid overdose; after removing these two the positive predictive value was 88%.

**Conclusions:**

These findings are consistent with our previous validation studies and suggest the computer case definition of sudden cardiac death is a useful tool for pharmacoepidemiologic studies of opioid analgesics.

## Findings

Identification of medications that increase the risk of sudden cardiac death has been a long-standing interest of pharmacoepidemiologists [1]. To facilitate the use of automated databases for these studies, we developed and validated a computerized case definition for this endpoint that had a positive predictive value between 86% and 88% [2]. As a result of the controversy regarding the cardiac safety of propoxyphene [3,4], there is interest in applying this definition to populations of opioid users. However, the patients in our previous validation study included a limited number of opioid users, for whom the information available in automated databases may be insufficient to distinguish out-of-hospital overdose deaths from sudden cardiac deaths. Thus, as part of an ongoing study of the cardiac safety of propoxyphene relative to hydrocodone, we validated our previously developed definition in a cohort of users of these two opioid analgesics.

## Methods

### Cohort

This validation study is part of an in-progress cohort study of persons with filled prescriptions for propoxyphene or hydrocodone between 1/1/1992 through 12/31/2007. The study was conducted with data from Tennessee Medicaid, computerized state hospital discharge records, and computerized state death certificate files [5-7]. As in our previous study [2], the cohort consisted of Medicaid enrollees 30-74 years of age. It was restricted to persons 30 to 74 years of age because for younger persons sudden cardiac death is very rare and may have a different etiology [8], and for older persons we found death certificates to be less reliable for identifying sudden cardiac deaths (unpublished data). Cohort members could have no life-threatening non-cardiovascular illness, diagnosed drug abuse, residence in a nursing home in the past year, or hospital stay within the past 30 days. Because we rely on Medicaid files to determine if inclusion/exclusion criteria are met, cohort membership required 365 days of Medicaid enrollment. We also required that cohort members have regular use of medical care, given our reliance on medical care encounters (physician diagnoses or prescribed medications) to determine cohort eligibility. Thus, cohort members had to have both outpatient encounters and filled prescriptions during the year preceding cohort entry.

### Study deaths

We identified cohort deaths that occurred following filling of each study opioid prescription, a time during which the patient was likely to have taken the opioid. This period was usually 30 days, but ended earlier if there was a subsequent study opioid prescription fill or if cohort eligibility criteria ceased to be met. Deaths were identified during this period that met our previously validated definition for sudden cardiac death [2]. As in our previous study [2], qualifying deaths had 1) no evidence of a terminal hospitalization, 2) underlying cause of death code consistent with sudden cardiac death, and 3) no terminal procedures inconsistent with unresuscitated cardiac arrest. To reduce the time and expense of record retrieval, the validation study was restricted to deaths in counties within a 100 mile radius of Nashville.

### Record retrieval and adjudication

For each study death, we abstracted the records of medical care encounters (hospital, emergency department, emergency medical services) around the time of death and obtained the medical examiner reports and copies of the paper death certificates when available. All pertinent information in the medical records was copied and redacted to remove personal identifiers. Each record was adjudicated by three study physicians (including a cardiac electrophysiologist). Cases for which there was any doubt in the adjudication process were reviewed by all of the investigators.

### Clinical definition of sudden cardiac death

The clinical definition was sudden cardiac death occurring in a community setting [9-12]. This was defined as a sudden pulseless condition (arrest) that was immediately fatal and was consistent with a ventricular tachyarrhythmia occurring in the absence of a known noncardiac condition as the proximate cause of the death [11]. Sudden cardiac deaths were classified as either probable or possible. *Probable sudden cardiac deaths* included a witnessed, sudden collapse with no pulse and respiration (or agonal), or an unwitnessed collapse in a person known to be alive within the previous hour, or ventricular fibrillation/tachycardia as the initial rhythm in cardiopulmonary resuscitation, or autopsy findings excluding causes other than a ventricular tachyarrhythmia. *Possible sudden cardiac deaths* were those in which no arrest was witnessed and the person was found moribund or dead, but with evidence that the subject had been alive in the preceding 24 hours. Both definitions excluded deaths from arrests that occurred in a hospital or other institutional setting, that were not sudden, or with documentation suggesting an underlying noncardiac cause (e.g., substance overdose or pneumonia) or a different cardiac etiology (e.g., heart failure or bradyarrhythmia).

The Vanderbilt Committee for the Protection of Human Subjects and the Tennessee Bureau of Medicaid and Department of Health approved the study.

## Results

**Figure 1:**
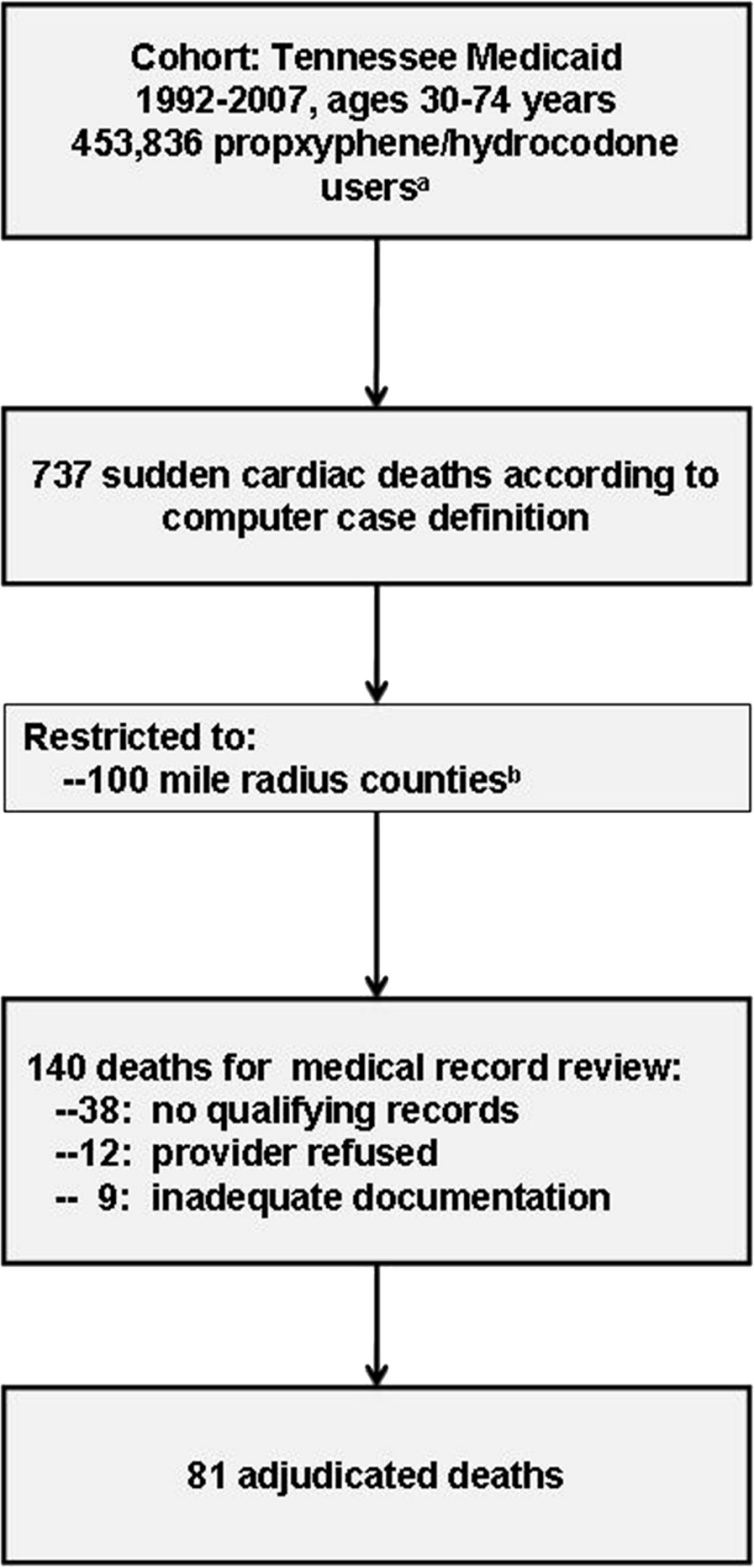
** Identification of potential sudden cardiac deaths subsequently adjudicated by medical record review. **^a^Cohort, includes 134,479 persons with propoxyphene prescriptions (last prescription during follow-up) and 319,357 with hydrocodone. ^b^Counties were Benton, Cheatham, Coffee, Davidson, DeKalb, Dickson, Hickman, Marshall, Maury, Montgomery, Putnam, Robertson, Rutherford, Smith, Sumner, Warren, Williamson, and Wilson.

The cohort included 453,836 persons with at least one qualifying prescription for propoxyphene or hydrocodone (Figure 1). There were 737 deaths that met the computer case definition for sudden cardiac death in the entire cohort. Of these, 140 occurred in the counties included in the validation study, of which 81 were adjudicated. The reasons for non-adjudication (Figure 1) include absence of terminal medical care encounters for which records could be retrieved (n = 38), provider refusal (n = 12), or insufficient information in the records for adjudication (n = 9).

Of the 81 adjudicated deaths, 85% had been coded according to the ICD10 classification system. The mean age at the time of death was 56 years, 49% were female, 69% resided in Standard Metropolitan Statistical Areas, and 52% had Medicaid enrollment as the result of disability. The records available for adjudication included either the medical examiner’s report or emergency department/emergency medical services records for all but 1 death. There were medical examiner’s reports for 42 cases, autopsy findings for 20 cases and reported levels of drugs for 26. We also obtained copies of the paper death certificates (that occasionally have physician notes not entered into the computerized files) for 80 of the adjudicated deaths.

Seventy three (90.1%) cases were adjudicated as probable or possible sudden cardiac deaths (Table 1). For two of these, post-mortem testing had identified levels of opioids or metabolites possibly consistent with death due to poisoning or overdose. Thus, 71 adjudicated cases (87.7%) were sudden cardiac deaths with no recorded evidence of poisoning or overdose. Of these, 69 (85.2%) were adjudicated as probable cases and 2 (2.5%) as possible cases.

**Table 1:** Performance of the computer case definition for sudden cardiac death

	**N**	**%**
Adjudicated cases	81	100.0
Not sudden cardiac death^a^	8	9.9
Sudden cardiac death	73	90.1
Evidence potential opioid overdose^b^	2	2.5
No evidence opioid overdose	71	87.7
Probable sudden cardiac death	69	85.2
Possible sudden cardiac death	2	2.5

^a^The excluded cases include: 2, bradycardia with weak pulse; 1, pulseless electrical activity; 1, concurrent myocardial infarction/motor vehicle crash; 1, congestive heart failure; 1, pneumonia; 1, witnessed gradual onset death; and 1, respiratory failure with hypotension.

^b^Based on post-mortem tests for opioid metabolites possibly consistent with overdose.

## Discussion

We sought to validate a previously developed computer case definition for sudden cardiac death in a population of opioid users. Although previous studies found that the definition had a positive predictive value of between 86% and 88% [2], it had not been specifically studied in a population of opioid users. We were concerned that the definition might not perform as well in this population, given that out-of-hospital overdose deaths might plausibly be confused with sudden cardiac deaths.

However, for persons with a propoxyphene/hydrocodone prescription filled within 30 days of death, the performance of the case definition was entirely comparable to that found in the prior study. The positive predictive value was 90% overall and 88% after exclusion of cases with evidence in the medical record of possible overdose. The high positive predictive value may be due in part to the study of a population in which out-of-hospital deaths should be infrequent. The maximum age for the underlying cohort was 74 years and nursing home residents or persons with life-threatening illnesses were excluded. For these patients, out-of-hospital deaths may be likely to trigger further investigation. Indeed, more than 50% of study deaths had a medical examiners report. This suggests caution with regard to extrapolation of study findings to higher risk populations in which such deaths are less likely to be unexpected and thus less frequently investigated.

The cohort excluded persons with prior medical encounters indicating possible drug abuse. We did this because of concern that such persons might have elevated risk for overdose deaths. If the study definition is utilized for this population, further validation would be prudent.

The study sample consisted of persons recently prescribed two specific opioids, one of which has been withdrawn from the market. However, the key study finding--minimal misclassification of opioid overdose deaths as sudden cardiac deaths--should have applicability to other drugs in this class, given their similar pharmacologic properties. Nevertheless, further studies in patients taking other opioids would be useful.

Of sample deaths, 42% were not adjudicated because of provider refusal/inadequate documentation (15%) or because there was no autopsy or record of terminal medical care (27%). We believe the former are related to factors that are unlikely to materially affect study findings, such as provider convenience and quality of witness reports. However, the latter are of greater concern, particularly if deaths for which there is no terminal care/investigation differ from the other deaths. It is unclear how this might affect the positive predictive value. The absence of terminal care/investigation might reflect rapidity of death or social factors--such as living alone. These would seem unlikely to alter the positive predictive value. Conversely, some of these patients might have had a terminal illness not recorded in the Medicaid encounters, in which case the positive predictive value would be lower.

Our study had several other limitations. The study did not include persons 75 years of age or older and it consisted of Tennessee Medicaid enrollees, of whom more than half had disability-related enrollment. These population restrictions may limit generalizability. The validation sample only included deaths that met the computer case definition and thus cannot be used to estimate sensitivity. Further studies that addressed these limitations would be useful.

In conclusion, a previously developed computer case definition for sudden cardiac death had a positive predictive value between 88% (excluding 2 possible overdoses) and 90% (including the overdoses) in a population of propoxyphene and hydrocodone users. These findings, consistent with our previous validation studies, suggest the computer case definition is a useful tool for pharmacoepidemiologic studies of opioid analgesics.

## Competing interests

Supported in part by a contract from the Food and Drug Administration (HHSF223201000011I), the National Heart Lung and Blood Institute (grant HL081707) and the National Institute for Arthritis and Musculoskeletal and Skin Diseases (grant P60AR056116). The views expressed are the authors’ and not necessarily those of the Food and Drug Administration. The authors declare that they have no competing interests.

## Authors’ contribution

The listed authors were entirely responsible for study design, data analysis, manuscript preparation, and publication decisions; no other persons were involved. All authors read and approved the final manuscript.
